# Dr. Cuming's Case of Tinea Capitis

**Published:** 1806-01

**Authors:** Ralph Cuming

**Affiliations:** H. M. S. Pegasus


### *To the Editors of the Medical and Physical Journal.*

Gentlemen,

FROM the manner in which Mr. Blackburn announces his mode of cure in tinea capitis, through the medium of your publication, it seems very evident, that he considers his remedy an infallible one; and that it may prove as successful in the hands of others, as it appears to have done in his, is my most sincere wish. The advantages accruing from a medicine so simple and mild in its operation, are extremely obvious; but in case of its failure, I beg leave to recommend the following method, which I believe will generally be found efficient, and which I shall illustrate by a brief recital of the case of a patient who was lately cured by me, extracted out of *my* Medical and Physical Journal.

Alexander Harris, twelve years of age, had been affected with tinea capitis for the space of four or five years, and a variety of applications, such as pitch plasters, &c. were used, without any evident effect during the above period. On my joining this ship, he was presented to me for the cure of it, which I set about by ordering his head to be shaved, and the spots to be well rubbed with ung. hydrarg. nitrati. This treatment was persevered in for some time, and his head shaved as occasion required, but the cure did not advance with that celerity which I could have wished. I therefore, on the 18th of October, had the whole of his head blistered; and after the cuticle was removed, the parts which appeared white, and formed the seat of the disease, were well stimulated with emplastrum epispasticum, as long as the whiteness appeared, which was from time to time rubbed off by means of tow. During the application of the cantharides, the little fellow was seized with strangury, which was cured by omitting the acrid application, and the administration of the following mixture:

℞, Pulv. gum. arab. ℨiij, salis nitri ℨij. tinct. opii gt. 40, syr syr. simp. ℥ß. M. ft. mist. capiat cochl. ampla ij. tertiâ quâque horâ donec fluit copiose urina.

24th, The spots which shewed marks of the disease, were lightly sprinkled over with hydrargyrus nitratus ruber, bene pulverizatus, covered with emollient dressings, and in a few days the whole of the sores were healed without leaving any marks of the disease.

Nov. 10. Three small white specks made their appearance, on which blistering plasters were applied, and the vesicated parts treated as above, which in a few days effected a perfect cure. I was apprehensive that the places which had undergone this medical discipline would have remained bald; but on placing the patient between the light and myself, I discovered a lanuginous appearance, which has been gradually acquiring strength; his head remains without spot or blemish, and I have no doubt will continue to do so. The plan which in this instance proved successful, in eradicating this loathsome disease, is certainly attended with some degree of pain; but it must be remembered, that obstinate diseases are not to be always cured by lenient means, and that professional men have often been foiled in their attempts to perform a radical cure, being finally compelled to yield the palm of victory to the nostrum-monger and Charlatan, which should operate as a caution, and induce them to leave no remedy untried.

I have lately had a bad case of trismus under my care, which I have reason to suppose was occasioned by the effects of cold; the patient at present is in a fair way of recovery; should any of your Correspondents have witnessed a similar case, they will oblige me by giving a history of it. This man had a slight cut in the palm of one of his hands, which I think did not penetrate through the cutis vera, and which was healed several days prior to his seizure with trismus; but as he had none of those symptoms which I have observed were the constant concomitants of this dreadful disease, when originating from punctured and other wounds, I am firmly of opinion, that my patient's complaint could not be attributed to this cause.

I am, &c.

RALPH CUMING, M. D.

*H. M. S. Pegasus*, Dec. 10, 1805.

CASE

